# Role of Melatonin in Plant Tolerance to Soil Stressors: Salinity, pH and Heavy Metals

**DOI:** 10.3390/molecules25225359

**Published:** 2020-11-17

**Authors:** Mohamed Moustafa-Farag, Amr Elkelish, Mohamed Dafea, Mumtaz Khan, Marino B. Arnao, Magdi T. Abdelhamid, Aziz Abu El-Ezz, Abdlwareth Almoneafy, Ahmed Mahmoud, Mahrous Awad, Linfeng Li, Yanhong Wang, Mirza Hasanuzzaman, Shaoying Ai

**Affiliations:** 1 Institute of Agricultural Resources and Environment, Guangdong Academy of Agricultural Sciences, Guangzhou 510640, China; lilinfeng@gdaas.cn (L.L.); wangyanhong@gdaas.cn (Y.W.); 2 Agriculture Research Center, Horticulture Research Institute, 9 Gmaa St, Giza 12619, Egypt; mohameddafea@yahoo.com (M.D.); 11716103@zju.edu.cn (A.M.); 3 Botany Department, Faculty of Science, Suez Canal University, Ismailia 41522, Egypt; amr.elkelish@science.suez.edu.eg; 4 Directorate of Regional Services, Allama Iqbal Open University, Islamabad 44000, Pakistan; mkhan@gu.edu.pk; 5 Department of Plant Physiology, Faculty of Biology, University of Murcia, 30100 Murcia, Spain; marino@um.es; 6 National Research Centre, Botany Department, 33 EL Bohouth St., Dokki, Giza 12622, Egypt; mt.abdelhamid@nrc.sci.eg; 7 Rice Research & Training Center, Agricultural Research Center, Giza 12619, Egypt; abuelezz76@hotmail.com; 8 Department of Biology Sciences, College of Education and Science at Rada’a, Albaydaa University, Rada’a, Yemen; std2008@gmail.com; 9 Laboratory of Germplasm Innovation and Molecular Breeding, Institute of Vegetable Science, Zhejiang University, Hangzhou 310058, China; 10 Department of Soils and Water, Faculty of Agriculture, Al-AzharUniversity, Assiut 71524, Egypt; mahrousawad.4419@azhar.edu.eg; 11 Department of Agronomy, Faculty of Agriculture, Sher-e-Bangla Agricultural University, Dhaka-1207, Bangladesh; mhzsauag@yahoo.com

**Keywords:** acidity, alkalinity, antioxidants, heavy metals, melatonin, salinity

## Abstract

Melatonin (MT) is a pleiotropic molecule with diverse and numerous actions both in plants and animals. In plants, MT acts as an excellent promotor of tolerance against abiotic stress situations such as drought, cold, heat, salinity, and chemical pollutants. In all these situations, MT has a stimulating effect on plants, fomenting many changes in biochemical processes and stress-related gene expression. Melatonin plays vital roles as an antioxidant and can work as a free radical scavenger to protect plants from oxidative stress by stabilization cell redox status; however, MT can alleviate the toxic oxygen and nitrogen species. Beyond this, MT stimulates the antioxidant enzymes and augments antioxidants, as well as activates the ascorbate–glutathione (AsA–GSH) cycle to scavenge excess reactive oxygen species (ROS). In this review, we examine the recent data on the capacity of MT to alleviate the effects of common abiotic soil stressors, such as salinity, alkalinity, acidity, and the presence of heavy metals, reinforcing the general metabolism of plants and counteracting harmful agents. An exhaustive analysis of the latest advances in this regard is presented, and possible future applications of MT are discussed.

## 1. Introduction

Melatonin (MT; *N*-acetyl-5-methoxytryptamine) is an indoleamine known to have multiple functions in humans and animals. Melatonin was then discovered in plants in 1995 [1,2], where it has a multitude of regulatory functions [3,4]. In mammalian, MT regulates seasonal changes at different levels of neuroendocrine and physiological functions [5,6], which affects circadian rhythms [7] and also shows a hypnotic effect. It plays a role in sleep initiation, vast regulatory activity, immunomodulation, and the inhibition of dopamine release from the retina [8,9].

Melatonin is an ecofriendly biomolecule that can penetrate cell compartments because of its small size and a high degree of solubility in both water and lipids. The use of MT is considered an alternative and inexpensive strategy to improve plant tolerance against abiotic stressors such as salinity, pH, and heavy metals. Phytomelatonin is synthesized from tryptophan under the activation of several enzymes [10]. The enzyme of tryptophan decarboxylase (TDC) first catalyzes 5-hydroxytryptophan to serotonin or tryptophan into tryptamine in the phytomelatonin biosynthetic pathway [10]. Then, the enzyme tryptophan 5-hydroxylase (T5H) catalyzes tryptophan to 5-hydroxytryptophan, and *N*-acetyl tryptamine to *N*- acetyl serotonin reactions. After that, serotonin *N*-acetyltransferase (SNAT) catalyzes the movement of the acetyl group from acetylcoenzyme A to different biomolecules. Lastly, phytomelatonin is synthesized through catalysis of *N*-acetylserotonin via the 5-hydroxyindol *O*-methyltransferase enzyme [10].

Melatonin acts as an effective antioxidant against both of reactive oxygen species (ROS) and reactive nitrogen species (RNS). Moreover, melatonin is a protective agent against different abiotic stresses [3,11,12]. Although each stressful agent provides concrete details in the induced physiological responses, MT, in general, reinforces physiological processes such as stomatic uptake, growth, rooting, germination, photosynthesis, osmoregulation, anti-senescence, primary and secondary metabolism, and plant hormone regulation [3,13]. Moreover, MT induces numerous changes in gene expression. These regulatory changes are beneficial for dealing with adverse situations and providing reinforcement against plant stress. There is hardly any review discussing the role of MT on multiple soil stressors. In this work, we provided an extensive review of the protective role of MT against several soil stressors such as salinity, pH (acidity and alkalinity), and the presence of heavy metals. These stressors are analyzed and discussed separately according to the methods or techniques used to combat them, and also the solutions that through the possible use of MT are elucidated according to current data. The possible mechanism of action to induce plant stress tolerance in each case is also presented, and suggestions are made concerning future expectations included for each stressor studied.

## 2. Salinity Stress Impacts and Tolerance in Plants

### 2.1. Plant Responses and Tolerance to Salinity Stress

Salinity is one of the environmental factors that threaten agricultural production, affecting more than 800 million ha worldwide [14]. The negative impacts of salinity reported for the different stages of plant growth include a reduction in photosynthetic activity, changes in carbohydrate and protein metabolism, while the accumulation of organic acids and osmolytes is the means of plant response to salinity stress [15,16]. The first biochemical sign of salinity is the generation of ROS [17,18,19], their harmful effects such as protein degradation, DNA mutation, and lipid peroxidation [20,21], which result in oxidative damage and the down-regulation of CO_2_ fixation, leading to physiological dysfunctions and programmed cell death [22,23,24]. Salinity reduces the germination percentage [25], cell expansion and plant growth and speeds up leaf senescence, adding to losses in yield [26]. 

Salinity causes alteration and imbalances in the nutrient content, as well as their partitioning within the plant [27]. In addition, the content of sodium (Na^+^) and chloride (Cl^−^) is increased under saline conditions, which leads to ion toxicity [28]. Na^+^ reduces calcium and potassium (K^+^) uptake and their transport to growing parts, while Cl^−^ reduces nitrate uptake, a combination of complex interactions that affect the plant metabolism and susceptibility to injury [29].

Plants improve their tolerance to salinity through decreasing salt accumulation as they reduce salt transport to aerial parts, ion compartmentation, osmotic adjustment, and the induction of antioxidant enzymes [14]. Many approaches have been adopted to overcome salinity, including soil reclamation programs, which probably represents the most effective and long-lasting method to minimize the hazards of salinity [30,31]. Fertilization can contribute to increasing salinity problems as fertilizers are a source of salts; for this reason, it is necessary to adopt suitable fertilization strategies [32], as well as undertake soil amendment and bio-inoculation, and apply leaf nutrients and mineral acids. Other agricultural practices, such as irrigation and drainage, and techniques such as grafting can also be modified to reduce salinity [31]. Recently, various exogenous protectants, such as phytohormones, signaling molecules, osmolytes, anti-oxidants, among others, have been extensively used to enhance plant tolerance to salinity stress [16,21].

### 2.2. Melatonin and Salinity Stress

Melatonin is known for its anti-oxidative potential, and recently the regulatory role of MT to enhance plant tolerance to different types of abiotic stress, including salinity, has been documented [33]. Exogenous applications of MT have been seen to improve the antioxidant system, protect cell membranes and enhance under saline conditions in tomato (*Solanum lycopersicum)* [34,35], cucumber (*Cucumis sativus)* [36], and watermelon (*Citrullus lanatus)* [37]. In barley (*Hordeum vulgare)* roots, the content of MT increased over control in response to NaCl and ZnSO_4_, an increase that plays a significant role in stress tolerance [3]. Moreover, MT inhibits stomatal closure [37], protects chlorophyll [36], and improves light absorption, CO_2_ fixation, and photosynthetic activity. Melatonin application increases the accumulation of organic osmolytes, including soluble sugars, water-soluble protein, and proline, thus protecting cells from dehydration under salt stress [38]. Furthermore, MT was seen to enhance ion homeostasis in *Malus hupehensis* under high-salinity conditions [33] and reduce ion toxicity by decreasing Na^+^ and Cl^-^ uptake [38]. It also regulates energy production, leading to the enhancement of germination and greater uniformity of salt-stressed cucumber seeds [39]. It has been shown that MT not only reduces the abscisic acid (ABA) content but also increases the content of gibberellins and indole-3-acetic acid, plant hormones that play significant roles in many biological processes in saline conditions [28]. The research carried out on the use of MT to alleviate salinity stress in different plants is summarized in Table 1.

**Figure 1:**
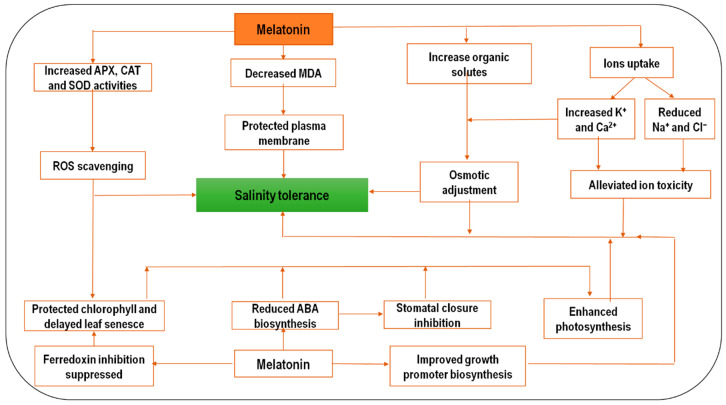
A schematic summary of the physiological responses of melatonin employed in salinity stress tolerance (based on available research findings). The impact of salinity in several physiological reactions such as reactive oxygen species (ROS), abscisic acid (ABA), sodium and chloride ions, and stomatal closure. Additionally, melatonin promotes plant tolerance to salinity stress by enhancing several pathways such as membrane integrity, chlorophyll, photosynthesis, plant growth, and potassium and calcium ions.

Melatonin plays various roles that protect plants against salt stress by inhibiting oxidative stress (Figure 1) [46]. The exogenous application of MT leads to the accumulation of endogenous MT under salinity stress, in wheat, by increasing the *TaSNAT* transcript, which encodes key enzymes in the MT biosynthesis pathway [47]. Under salinity stress, MT upregulates the expression of antioxidant-related genes. For instance, MT was reported to increase the ascorbate peroxidase (APX), catalase (CAT), and superoxide dismutase (SOD) activities in salt-stressed *Arabidopsis* by upregulating *APX1/2, CAT1*, and *FSD1* transcripts [48]. In addition, it upregulated genes involved in ascorbate metabolism, including *VTC4* and *APX4*, under salt-stress conditions. This may explain the impact of MT in promoting the antioxidant capacity of plants [44].

Melatonin protects the photosynthetic machinery from salt-induced oxidative damage [49]. It inhibits ROS accumulation in leaves of salt-sensitive cucumber plants by enhancing antioxidant enzymes [36]. Exogenous MT suppresses chlorophyll degradation in rice leaves [40]. It suppresses salt inhibition of the ferredoxin gene *PetF* in rice [50], while ferredoxin protects chlorophyll from degradation in rice [51]. Melatonin was seen to protect the total chlorophyll content and alleviate the salt-induced decrease in the net photosynthetic rate, and the maximum quantum efficiency of photosystem°II°(PSII) of cucumber [36]. The MT-mediated protection is closely associated with the inhibition of stomatal closure and improved light energy absorption and electron transport in photosystem II in *Mentha × piperita* and *Menthaarvensis* plants [52]. Furthermore, MT delays leaf senescence in rice [40]. 

Melatonin may maintain the integrity of biological membranes, improving the permeability and reducing lipid peroxidation; both of these alleviate toxicity and enhance plant growth in maize seedlings [42]. The observation that the addition of MT decreases malondialdehyde (MDA) levels in cucumber confirms that MT can protect biological membranes against salt-induced damage [36]. Energy production is an integral part of the mechanism of MT that alleviates the detrimental impact of salinity; proteomic analysis of salt-stressed cucumber germinating seeds revealed that many enzymes involved in ATP production were upregulated in response to exogenous MT application [39]. Similarly, MT helps plants to increase the energy generated from lipids stored in sweet potato cells, and a good energy status is necessary for the maintenance of proton pump activity across the tonoplast and plasma membrane [53].

Melatonin possibly improves salinity tolerance by upregulating the expression of ion-channel genes in leaves such as *MdNHX1* and *MdAKT1*, contributing to the maintenance of ion homeostasis [54]. The exogenous application of MT increased the potassium content, whereas the Na^+^ content was significantly reduced [42]. Added MT reduced Na^+^ and Cl^-^ accumulation in roots and leaves of both salt sensitive and tolerant rice seedlings, an effect that was associated with the upregulated transcription of *OsSOS1* in roots and of *OsCLC1* and *OsCLC2* in roots and leaves [41]. The increased K^+^ and Ca^++^ content of salt-stressed plants in response to MT application may improve the salt tolerance of plants, reducing Na^+^ uptake and accumulation, particularly in leaves [28].

Recent reports indicate that MT does not act alone in the amelioration of salinity stress. For example, it increases the accumulation of endogenous bioactive molecules known for their salt-stress mitigation role. Melatonin was seen to accelerate polyamine biosynthesis from precursor amino acids, and decrease the salt-induced degradation of polyamines [47]. Melatonin also improved the gibberellin content and ABA degradation and thus enhanced the metabolism in salt-stressed germinating seeds [45]. In another study, Zhao et al. [55] observed, downstream MT, an increase in the endogenous NO content in alleviating salinity stress. Exogenously applied MT enhanced seed germination under salt stress, an observation that was associated with the upregulation of gibberellins biosynthesis genes (e.g., *GA20ox* and *GA3ox*) and ABA catabolism genes (e.g., *CsCYP707A1* and *CsCYP707A2*), while ABA biosynthesis genes (e.g., *CsNECD2*) were downregulated [45,56].

## 3. pH Stress

### 3.1. Impact of pH Stress and Tolerance in Plants

Soil pH (potential of hydrogen) is a vital growth factor that directly affects plant growth and development, soil mineral solubility, and soil leaching [57,58,59]. The optimal pH for crop production ranges from 6 to 8 [60]. However, pH stress may occur in alkaline soils, those with a high pH (>9), and acid sulfate soils; soils in drained coastal wetlands suffer from extremely low pH (<4) because the sulfur present in the sediment may be oxidized to sulfuric acid or due to the oxidation of pyrite [61]. Sodic alkaline stress results from soils having a high Na_2_CO_3_ or NaHCO_3_ content, while alkaline soils are characterized by a high pH in the rhizosphere in a low fertility soil with low water content [62,63]. As pH affects sustainable crop production, developing soil conditioners and adapting agricultural practices to mitigate the effects of extreme soil acidity and alkalinity are crucial for both soil quality conservation and productivity. For instance, alkaline stress causes a metabolic imbalance in plants, which leads to oxidative stress and damage of nucleic acids and proteins [63] as well as chlorophyll degradation and a reduction in photosystem II efficiency, which results in the overall failure of photosynthesis [64,65]. On the other hand, acidic soils also limit crop growth and productivity [66] and may become a severe problem due to the overuse and misusage of chemical fertilizers, especially nitrogen fertilizers, and increasing the heavy metal solubility. Acid rain has a similar effect [67,68]. In acidic soils, weak crop growth and yield generally result from the combination of toxicity caused by hydrogen, aluminum, and manganese, and a deficiency of nutrients such as phosphorus, calcium, magnesium, potassium, and molybdenum, as well as a reduction in water absorption [66,68].

Several methods and techniques have been applied to alleviate pH stress, such as adding soil amendments or organic materials, and planting tolerant cultivars in acidic soil, although, in alkaline soils, a variety of physical, chemical, and agricultural practices have been used.

In acidic soils, liming is one of the best standard solutions that directly and indirectly correct soil acidity and enhance agricultural productivity [69] by increasing the calcium and magnesium content of the soil. This increases soil pH and thus increases the availability of phosphorus and molybdenum [70]. Liming materials also improve the efficiency of nitrogen uptake and enhances nodule formation in legumes in acidic soils [71,72]. Finally, many reports found that liming enhances root growth in annual crops [70]. Additionally, gypsum is a cheap amendment that improves plant growth by leaching and eliminating soil acidity [73]. The cultivation of a tolerant plant species in acid soils is also a right approach, although it must be borne in mind that the tolerance to soil acidity not only varies among crop varieties but also genotypes within a species [74,75]. Most of the plant species tolerant to acidity have their center of origin in acid soil regions, indicating that adaptation to soil limitations belongs to evolutionary processes [76,77]. Several organic materials have also been applied to improve soil acidity; for instance, peat moss, plant residues, and organic manures. The pH of such material should undergo surface modification to confirm its natural or partial alkalinity [78,79]. The inclusion of waste plant materials to acidic soils significantly reduces Al saturation, raising soil pH, and enhancing the plant growth profile.

In addition, several physical, chemical, and soil management strategies are applied to tackle the negative impact of alkaline soils on large-scale crop production [80,81]. The physical methods include soil leaching, bringing the salts out of the soil after dissolving them, soil scratching, and water discharge [82,83]. For their part, chemical methods involve applying elements that assist in the removal of exchangeable sodium from the soil surface [84]. The point is that exchangeable sodium exists in different quantities; thus, the leaching of sodium should be undertaken. The chemical materials used can be categorized as soluble calcium salts (gypsum and phospho gypsum), poorly soluble calcium salt (limestone), and acid-producing compounds (e.g., sulphur, sulphuric acid, pyrites) [81,84,85]. Besides physical and chemical methods, soil management practices can mediate alkaline stress [86,87]. In general, these include an increase in the organic matter, preparation of the field, preparation of basin and sowing, crop rotation, the use of carbonic material and fertilizers (zinc, iron, manganese, and nitrogen), and finally, growing crops that bear salts and alkaline tolerance [85,88].

### 3.2. Melatonin and pH Stress

Former reports on MT with pH stress are shown in Table 2. With the discovery of MT in plants, studies on MT started to increase sharply [89]. It was found that pH stress can increase endogenous MT levels, and some reports mention that they may reach 12 times the level found in untreated plants [90,91,92,93]. There is little research on the amelioration effect of exogenous MT applied in the face of soil pH stress in plants. Liu et al. [94] found that MT improved the plant growth of tomato (*Solanum lycopersicum*) under alkaline and acid pH stress. The exogenous application of MT (0.1 and 1 μM) in *Glycine max* efficiently mitigated aluminum toxicity in an acidic soil by modulating anti-oxidative enzymes and enhancing organic acid anion exudation, thereby enhancing Al phytotoxicity [39].

**Figure 2:**
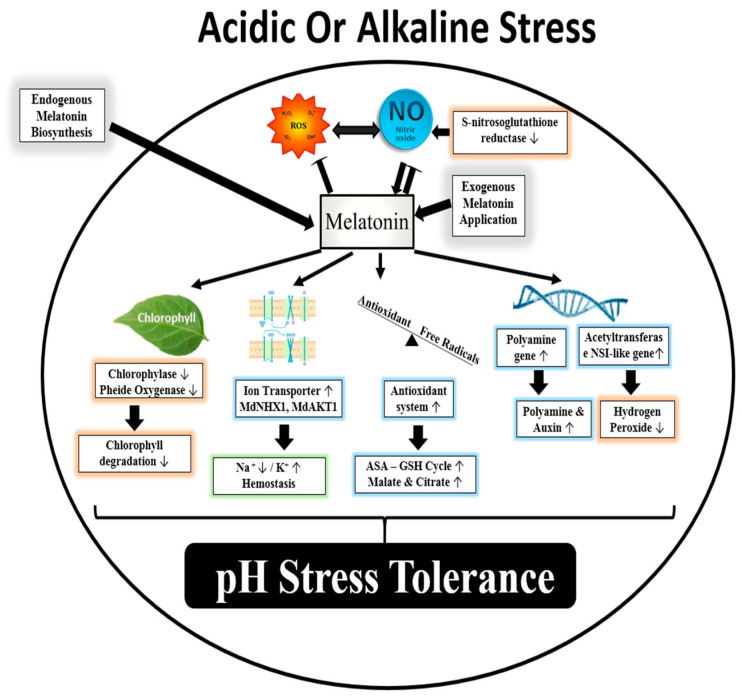
Model of melatonin action under acidity and/or alkalinity stress. Melatonin triggers the accumulation of auxin, polyamines, and nitric oxide. Furthermore, managing ion homeostasis by decreasing Na+ content and increasing K^+^ content. Melatonin reduced oxidative stress by triggering the antioxidant machinery and decreasing chlorophyll degradation. Exogenous melatonin induces NO generation, which subsequently upregulates the expression level of defense genes.

Many of the biological effects of MT under alkaline or acid conditions are generated via the activation of MT receptors (MTNR1A, MTNR1B), while others result from its role as an antioxidant, functioning as the first line of defense against oxidative stress [90,96]. Nevertheless, the limited number of papers addressing the mechanistic pathway followed by MT during pH stress means that its potential role in plants is unclear (Figure 2). Understandably, the first hypothesis concerning MT in plants suggested it had the identical features of those observed in mammals. Thus, the first experimental studies of the physiological role of MT in plants under pH stress tested its possible involvement in cell protection and reproductive and vegetative development. Nevertheless, it was found to be the critical role of MT and its shared action in the biosynthetic pathways of many phytohormones, especially auxin [44] (Figure 2). Moreover, it was found that MT influenced auxin accumulation and transport (through PIN transporters), and signal transduction through the NO signaling pathway (Figure 2). Besides, the treatment of alkaline-stressed seedlings with MT increased the accumulation of polyamines and the transcript levels of genes involved in (PA) synthesis [95] (Figure 2). In response to alkaline stress, MT reduced oxidative stress by triggering antioxidant scavenging activity, especially ascorbic acid and glutathione [97,98]. MT supplementation decreases membrane leakage and helps the plant regain its regular root architecture [95] (Figure 2). Such antioxidant machinery probably preserves and restores the grana lamella of the chloroplast, preventing chlorophyll degradation as a result of stress and improving photosynthesis [92]. Melatonin contributes to the maintenance of ion homeostasis by decreasing the Na^+^ content and increasing the K^+^ content. Other reports mention the protective effect of MT under sodic alkaline stress through NO signaling. Under pH stress in plant root, MT triggers the accumulation of endogenous NO by down-regulating the expression of *S-nitrosoglutathione* reductase [99]. This evidence strongly suggests that elevation of NO due to *S-nitrosoglutathione* reductase activity and auxin signaling was significantly correlated to the adventitious root formation by MT [100,101]. Confocal laser scanning microscopy and specific NO-sensitive fluorophores showed a high rate of NO accumulation in epidermal and xylem, while less intense rates of NO have been detected in the cortex in pea roots [102]. Such findings suggest that NO might serve as a downstream signal in plant tolerance to alkaline stress [94]. Similarly, in response to Al toxicity in acidic soils, MT significantly increases the expression of acetyltransferase NSI-like genes, lowering the production of hydrogen peroxide and increasing the exudation of malate and citrate from roots [103].

## 4. Heavy Metal Stress

### 4.1. Heavy Metal Stress: Impact and Tolerance in Plant

Heavy metals (HMs) are either non-essential or minutely required elements for normal plant growth and development. They are ubiquitously found in the soil–water environment and readily taken up by the plants, thereby causing oxidative stress [104]. Plants combat HM stress by inbuilt defensive mechanisms, the exogenous application of synthetic agents, or by enhancing plant tolerance through genetic modifications. Some inbuilt mechanisms include metal exclusion, restricted foliar translocation, metal sequestration and compartmentalization, chelation, and scavenging of free radicals by antioxidant enzymes [105]. Phytochelatins are cysteine-rich polypeptides that form complexes with HMs in the cytosol, followed by their storage in the vacuoles. Antioxidant enzymes scavenge free radicals and convert them into non-hazardous molecules, such as enzymes; several other molecules, such as metallothioneins, organic acids, phenols, and α-tocopherol, also contribute to the plant tolerance against HM stress [39,104]. Moreover, signaling pathways help plants in the perception of stress and activation of pathways involved in the calcium, mitogen, ROS, and hormones metabolisms [106,107]. The external application of several agents such as glutathione, hydrogen sulfide, salicylic acid, or priming with hydrogen peroxide (H_2_O_2_), MT, and nitroprusside enhance plant’s tolerance to HM stress [108].

The identification of genes associated with stress tolerance and the integration of these into the plant genomes are key strategies to enhance plant tolerance to HM stress. Research has shown that plants genetically engineered with HM-resistant genes have better chances of survival and better growth than untransformed plants; for example, *Brassica napus*, *Nicotiana tabacum* [109], *Arabidopsis thaliana* [110], and *Brassica juncea* [111] have shown increased tolerance to HM stress when genetically engineered.

### 4.2. Melatonin and Heavy Metal Stress

The use of MT to regulate plant growth under HM stress has been extensively studied [112,113]. However, growth regulation is dependent on plant species, metal concentration, and the applied dose of MT (Table 3). For example, Al-stressed soybean plants (50 μM) exhibited improved root growth, enhanced antioxidant activities, and root exudation when supplied with 1 μM MT, but 100 μM and 200 μM MT induced no response [103]. Under Cu stress, low exogenously applied MT level (10 μM) positively affected germination and growth in the red cabbage (*Brassica oleracea var. capitata f. rubra*) while higher levels (100 μM) had a negative effect [114]. In addition, under low MT treatment, Al-stressed soybeans demonstrated enhanced antioxidant activities and improved tolerance while higher doses negatively affected root growth [39]. On the contrary, the growth of tomato plants, affected by 100 μM Cd, was optimally regulated by a relatively high MT concentration of 100 μM [112]. Lead triggered cell death and morphological deformation in cultured tobacco (*Nicotiana tabacum*) provoked the bright yellow cell effect, which was reversed by MT supplementation [115,116]. However, Se, supplied as selenocysteine (3μM), improved MT levels in tomato plants treated with 100 μM Cd, leading to stabilized growth, reduced photoinhibition, and membrane leakage [117].

**Figure 3:**
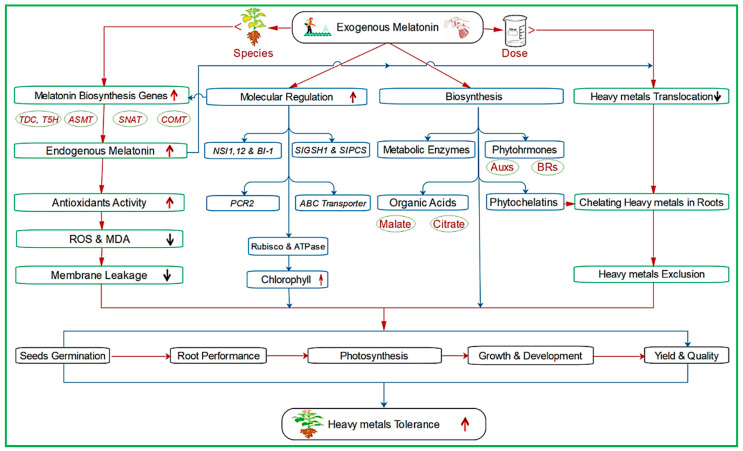
Melatonin induced mechanisms aimed at increasing plant tolerance to heavy metals. The positive effect of MT is species and dose-dependent responses. The exogenous MT induces the endogenous MT via upregulating the biosynthesis genes, which controls the ROS scavenging, molecular elements, biosynthesis, and heavy metal translocation. Moreover, MT regulates the molecular elements such as *NS* genes (nuclear shuttle protein-interacting), *Bl-1* (Bax inhibitor-1 protects against apoptosis), *SIGSH1* and *SIPCS* (responsible for GSH and PCS in tomato), ABC transporter and *PCR2* (stress-responsive genes), as well as *Rubisco and ATPase* (crucial genes to the photosynthesis). Besides, melatonin enhances the biosynthesis of metabolic enzymes, phytohormones (i.e., auxins (AUXs), and brassinosteroids (BRs)), organic acids (root exudates such as malate and citrate), and phytochelatins (chelate the heavy metals (HMs) in roots). Consequently, the whole plant life cycle is improved, starting with seed germination till yield and quality, collectively conferring heavy metal tolerance.

Melatonin provides multifaceted protection against HM stress in plants. It restricts the translocation of HMs and upregulates the involved genes in the MT biosynthesis pathways, thereby increasing internal MT levels to combat HMs stress (Figure 3; Table 4). ROS scavenging by MT in different plant species under HM stress has been reported previously [112,113]. ROS scavenging by MT involves several chemical reactions, including hydrogen donation, addition reactions, substitutions, and nitrosation. Structural analysis has revealed that the NH group in MT donates hydrogen ions and that indoleheterocycle is core to its antioxidant activity. Two side chains (*N*-acetyl and methoxy group) also aid in enhancing the antioxidant ability of MT [123]. In addition to direct ROS scavenging, MT enhances several antioxidant enzymes and other metabolic enzymes to improve plant tolerance [124,125]. For example, external MT enhanced the tolerance of wheat *(Triticum aestivum)* plants to ZnO nanoparticles by increasing Rubisco and ATPase activities, which are crucial to the photosynthesis [113]. In another study, Al-stressed soybean plants, treated with a low dose of MT, enhanced plant tolerance by modulating the activities of ROS scavenging enzymes, but higher doses had the opposite effect [103]. This suggested that the effects of MT on the activities of antioxidant enzymes in HM-stressed plants depend on the dose and plant species.

The increase in endogenous MT levels by external application or by genetic manipulation is also an important way of improving plant tolerance to adverse environmental conditions. Previous research has shown that exogenous MT application enhanced plant tolerance against HMs by increasing internal MT levels [126]. Similarly, genetic engineering has enabled plant biologists to manipulate internal MT biosynthesis and observe changes in its concentration under different biotic and abiotic stress conditions in higher plants. Exposure to HMs triggers the upregulation of genes involved in the MT biosynthesis pathway [39]. Similarly, the upregulation of MT encoding genes has been seen to enhance the activities of antioxidant enzymes in HM-stressed plants [127]. For example, silencing the heat shock factor A1a (*HsfA1a)* gene lowered Cd tolerance and MT levels in tomato plants, while its overexpression enhanced plant’s tolerance, accompanied by increased transcripts of the MT biosynthesis gene, caffeic acid *O*-methyltransferase 1 (*COMT1*). Further, when the *COMT1* gene was silenced in plants over-expressing *HSfA1a*, Cd tolerance was reduced due to less biosynthesis of MT [128]. In another study, *ZjOMT*, a methyltransferase-encoding gene cloned from *Zoysia japonica* was upregulated in shoots and roots of *Zoysia grass* under Al stress [129].

Melatonin triggers the biosynthesis of many plant hormones that regulate plant growth and development. For example, using RNAi technology in rice *(Oryza sativa*) plants, *SNAT2*, an isogene MT biosynthesis pathway gene, was silenced, leading to skotomorphogenesis, and suggesting a deficiency of brassinosteroids, which regulate plant growth under dark conditions or at night [130]. In another study, boron toxicity in spinach *(Spinacia oleracea)* was alleviated by MT-induced increase in indole acetic acid concentration [131]. MT is also involved in calcium signaling, which helps regulate plant growth in challenging environments [132].

Phytochelatin synthesis and restricted translocation of HMs to the foliar parts may also improve plant tolerance to the abiotic stresses. Previous research has shown that during HM stress, phytochelatin synthesis is reinforced in plants [112]. For example, [117] reported that external MT treatment reduced Cd accumulation and ROS generation in tomato leaves, but Cd concentration was high in the roots, suggesting chelation and compartmentalization of Cd in the root cells [112]. Further, under 50 mg·L^−1^ vanadium stress, watermelon (*Citrullus lanatus*) seeds, pre-treated with MT, produced plants with increased photosynthetic pigments [122]. As a résumé, Figure 3 depicts a model which compiles the most relevant agents and mechanisms that increase MT-mediated plant tolerance to HMs.

## 5. Summary and Conclusions

Abiotic stresses associated with soil, such as salinity, extreme pH values, or the presence of heavy metals, cause many problems in plants at physiological and molecular levels, resulting in enormous production losses worldwide. MT is a key bioactive molecule in the resistance of vascular plants to abiotic stress. In this review, we have summarized the role and mechanism of MT in increasing plant tolerance to soil-associated stress, especially its role as an antioxidant molecule. However, the door remains open for further research on MT and its impact in the face of salinity, extreme pH, and heavy metal stress. Regarding salinity, the anatomical modification of salt stressed in leaves and roots in response to MT application, and the impact of applied MT on salt-stressed plant pollen viability, fruit set, and abscission should be investigated. In addition, the possibility of eliciting seed populations and extensive plant crops to ensure homogenous and specific endogenous MT levels by each plant species, thus reinforcing stress tolerance response, is one of the most coveted goals in the search for seedlings and crops that are resistant in unsuitable soils.

In the case of pH stress, a deeper understanding of MT action under pH stress is still needed due to the diverse types and subtype of pH soil stress. The practical applicability of using MT in large-scale crop production in the face of alkalinity or acidity stress remains unconfirmed using viable concentrations, in that, many of the effects described have not been demonstrated in the field. Moreover, more intensive transcriptomic and proteomic analyses should help reveal the hidden pathway of MT in inducing alkaline and acidic stress tolerance. Furthermore, the accurate and precise determination of MT faces several challenges, and it is an essential task of future research to investigate the efficiency and safety concentration of MT in different stress situations. While significant advances have been made in establishing MT as a beneficial component of optimal plant growth, a great deal remains to be learned about the mechanisms involved, such as the mode and pathway of MT transport and its possible conjugated molecules under pH stress, as well as the interaction of MT with other phytohormones (auxin, gibberellin, cytokinin, abscisic acid, salicylic acid, and jasmonic acid), and its connection with growth, organogenesis, apical dominance, and tropisms under pH stress.

As for HM stress, we suggest that more efforts should be made to enhance endogenous MT levels in HM hyper-accumulator plants. The possible role of MT in alleviating stress induced by several radioactive elements is unknown and needs thorough investigation. It is known that MT interacts with primary and secondary metabolic pathways in plants, but the crosstalk with MT is unclear, and future research should also look at this issue. Finally, the modes of application of MT in phytoremediation strategies and the plant responses to each need to be studied in real field conditions.

## Figures and Tables

**Table 1:** Primitive impact of exogenous melatonin application on different salt-stressed plants.

Common Name	Scientific Name	Stress Treatment	MT Concentration	Findings	References
**Field crops**					
Rice	*Oryza sativa*	0.5% NaCl	0, 10, 20 μM	Antioxidants ↑, leaf senescence and cell death ↓, chlorophyll degradation ↓	[40]
Rice	*O. sativa*	150 and 200 mM NaCl	10–500 μM	Seed germination and root vigor ↑, antioxidant enzymes ↑, Na^+^ and Cl^−^ contents ↓	[41]
Maize	*Zea mays*	100 mM NaCl	1 μM	Antioxidant enzymes ↑, K^+^ contents and K^+^/Na^+^ ratios ↑, electrolyte leakage ↓, MDA ↓	[42]
Maize	*Z. mays*	150 mM NaCl	0–100 μM	Photosynthesis ↑, antioxidant enzymes ↑, Na^+^ contents ↓	[43]
Broad bean	*Vicia faba*	3.85 and 7.69 dSm^−1^ diluted seawater	0100 and 500 μM	Plant growth ↑, RWC ↑, photosynthesis ↑, carbohydrates ↑, phenolic content ↑, IAA ↑, K^+^,Ca^+2^, K^+^/Na^+^, and Ca^+2^/Na^+^ ratios ↑	[28]
Soybean	*Glycine max*	Soil saturated with 1% (*w*/*v*) NaCl	0–100 μM	Photosynthesis ↑, cell division ↑, carbohydrates ↑, fatty acid ↑, ascorbate ↑, the inhibitory effects on gene expressions ↓	[44]
Rapeseed	*Brassica napus*	0.75% NaCl	0–100 μM 30 μM	Antioxidant enzymes ↑, solute accumulation ↑	[38]
**Fruit crops**					
Pingyitiancha	*Malus hupehensis*	100 mM NaCl	0.1 μM	Photosynthesis ↑, ion homeostasis ↑, oxidative damage ↓	[33]
**Vegetable crops**					
Tomato	*Solanum lycopersicum*	75 mM NaCl	100 μM	Proteins and membranes protection ↑, antioxidants ↑, photosynthesis ↑	[34]
Tomato	*S.lycopersicum*	150 mM NaCl	0–200 μM	Photosynthesis ↑, ROS ↓	[35]
Cucumber	*Cucumis sativus*	150 mM NaCl	1 μM	Energy production regulation ↑	[39]
Cucumber	*C. sativus*	200 mM NaCl	0–200 μM	Antioxidant enzymes ↑, chlorophyll ↑, photosynthesis ↑	[36]
Cucumber	*C. sativus*	150 mM NaCl	(0–500 μM) 1 μM	GA3 biosynthesis ↑, germination rate ↑, ABA ↓, oxidative damage ↓	[45]
Watermelon	*Citrullus lanatus*	300 mM NaCl	50–150 μM	Photosynthesis ↑, antioxidant enzymes ↑, photosystem II efficiency ↑, stomatal closure ↓, oxidative damage ↓	[37]

Abbreviations: MT, melatonin; NaCl, sodium chloride; K^+^, potassium; MDA, malondialdehyde; RWC, relative water content; IAA, indole acetic acid; Ca^2+^, calcium; ROS, reactive oxygen species, GA_3_, gibberellic acid; ABA, abscisic acid.

**Table 2:** The action of melatonin in mitigation of pH stress responses.

Common Name	Scientific Name	Stress Type	MT Concentration *	Findings	References
Lupin	*Lupinus albus*	pH (3.5 to 8.5)	? **	↑Melatonin	[91]
Apple	*Malus hupehensis*	Alkalinity (pH 8.5 and 8.8)	5 μM	↑Polyamines, MDA ↓, ROS ↓, antioxidants ↑, polyamine synthesis genes↑	[95]
Tomato	*Solanum lycopersicum*	Acidity (pH 2.5)	100 μM	Photosynthesis ↑, antioxidants↑, ROS ↓	[92]

* Only the best doses of exogenous melatonin have been selected, which positively impacted plant tolerance against pH stress. ** No MT treatment. This report studied the possible changes in MT levels in response to different stressors, including pH.

**Table 3:** Role of melatonin in heavy metal stress tolerance.

Common Name	Scientific Name	Stress Concentration	MT Concentration *	Findings	Reference
**Cadmium**					
Wheat	*Triticum aestivum*	0.2 mM	50 μM	Antioxidants enzymes ↑	[118]
Alfalfa	*Medicago sativa*	50, 100, and 200 μM	50 μM	ABC transporter and PCR2 transcripts ↑, Cd accumulation ↓	[119]
Tree tomato	*Cyphomandrabetacea*	10 mg·L^−1^	50 μM	Antioxidants ↑, plant biomass ↑	[120]
Tomato	*Solanum lycopersicum*	25 and 100 μM	100 μM	Antioxidants ↑, glutathione and phytochelatins↑	[112]
Tomato	*Solanum lycopersicum*	100 μM	1 μM	Plant growth ↑, electrolyte leakage ↓, photoinhibition ↓	[117]
**Lead**					
Tobacco	*Nicotiana benthamiana*	15 μM	200 nM	DNA damage ↓, cell growth, and viability ↑	[116]
Tobacco	*Nicotiana benthamiana*	15 μM	200 nM	Cell proliferation ↑, cell death ↓	[115]
**Aluminum**					
Soybean	*Glycine max*	300 μM	100 mM	Antioxidants ↑, photosynthesis ↑, MDA ↓	[121]
**Copper**					
Red cabbage	*Brassica oleracea var. Capitata f. rubra*	0.5 and 1 mM	10 μM	Germination and fresh weight ↑, MDA ↓	[114]
**Vanadium**					
Watermelon	*Citrullus lanatus*	50 mg·L^−1^	0.1 μM	Plant growth ↑, chlorophyll ↑, photosynthesis ↑, antioxidant enzymes ↑, V accumulation ↓, ROS ↓, MDA ↓	[122]

* Only those maximum doses of exogenous melatonin have been selected, which had positive impacts on plant tolerance against heavy metal stresses. Abbreviations: Cd, cadmium; MDA, malondialdehyde; ROS, reactive oxygen species.

**Table 4:** Melatonin-upregulated genes under heavy metal stress.

Common Name	Scientific Name	HM Concentration	Melatonin Treatment *	Gene Name	Gene Description	Ref.
Wheat	*Triticum aestivum*	0.2 mM Cd	100 μM	*TaASMT1, TaASMT2* and *TaTDC*	*N*-acetylserotonin methyltransferase and tryptophan decarboxylase	[118]
Watermelon	*Citrullus lanatus*	50 mg·L^−1^ V	0.1 μM	Cla010664 and Cla004567	O-methyl transferase and methione *S*-methyl transferase	[122]
Tomato	*Solanum lycopersicum*	100 μM Cd	100 μM	*SIGSH1* and *SIPCS*	Responsible for GSH and PCS in tomato	[112]
Alfalafa	*Medicago sativa*	100 μM Cd	50 μM	*MsSNAT*	*M. sativa* Serotonin *N*-acetyltransferase (a melatonin synthetic gene)	[119]
Tomato	*Solanum lycopersicum*	100 μM Cd	NA	*HsfA1a* and *COMT1*	Heat shock factor A1a and caffeic acid O-methyltransferase 1	[128]
Zoysiagrass	*Zoysia japonica*	400 μM Al	NA	*ZjOMT*	An O-methyltransferase gene cloned from *Z. japonica*	[129]
Soybean	*Glycine max*	50 μM Al	1 μM	*NSI1* and *NSI2*	Genes encoding acetyltransferase NSI-like (nuclear shuttle protein-interacting)	[103]
Tomato	*Solanum lycopersicum*	100 μM Cd	1 μM	*TDC, T5H, SNAT, ASMT*	Melatonin biosynthetic genes	[117]
Tobaco	*Nicotiana tabacum*	15 μM Pb	200 nM	*BI-1*	Bax inhibitor-1 protects against apoptosis	[115]

* Only those maximum doses of exogenous melatonin have been selected, which had positive impacts on plant tolerance against heavy metal stresses. Cd (cadmium), Al (aluminum), V (vanadium).
